# Corrosion of Reinforced A630-420H Steel in Direct Contact with NaCl Solution

**DOI:** 10.3390/ma16176017

**Published:** 2023-09-01

**Authors:** Felipe M. Galleguillos Madrid, Alvaro Soliz, Luis Cáceres, Sebastian Salazar-Avalos, Danny Guzmán, Edelmira Gálvez

**Affiliations:** 1 Centro de Desarrollo Energético de Antofagasta, Universidad de Antofagasta, Av. Universidad de Antofagasta 02800, Antofagasta 1271155, Chile; sebastian.salazar@uantof.cl; 2 Departamento de Ingeniería en Metalurgia, Universidad de Atacama, Av. Copayapu 485, Copiapó 1530000, Chile; danny.guzman@uda.cl; 3 Departamento de Ingeniería Química y Procesos de Minerales, Universidad de Antofagasta, Av. Universidad de Antofagasta 02800, Antofagasta 1271155, Chile; luis.caceres@uantof.cl; 4 Departamento de Ingeniería Metalúrgica y Minas, Universidad Católica del Norte, Av. Angamos 610, Antofagasta 1270709, Chile; egalvez@ucn.cl

**Keywords:** A630-420H steel, mixed potential theory, oxygen reduction reaction, reinforced steel, saline solution

## Abstract

The deterioration of reinforced concrete structures in marine environments presents multiple problems due to the premature degradation of reinforced steel. This work aimed to study the corrosion of reinforced A630-420H steel when exposed to a 0.5 M NaCl solution. Although this carbon steel is the most widely used material for reinforced concrete structures in Chile, there is limited research on its resistance to corrosion when in contact with saline solutions. The electrochemical reactions and their roles in the corrosion rate were studied using linear sweep voltammetry, weight loss, scanning electron microscopy, and X-ray diffraction techniques. This analysis is unique as it used the superposition model based on mixed potential theory to determine the electrochemical and corrosion parameters. The outcomes of this study show that A630-420H steel has a higher corrosion rate than those of the other commercial carbon steels studied. This fact can be attributed to the competition between the cathodic oxygen reduction reaction and hydrogen evolution reaction, which also depends on the environmental conditions, exposure time, stabilization of the corrosion products layer, and presence of chloride ions. Additionally, the results under mechanical stress conditions show a brittle fracture of the corrosion product oriented longitudinally in the direction of the bend section, where the presence of pores and cracks were also observed. The corrosion products after corrosion were mainly composed of magnetite and lepidocrocite oxide phases, which is in concordance with the electrochemical results.

## 1. Introduction

**Figure 1:**
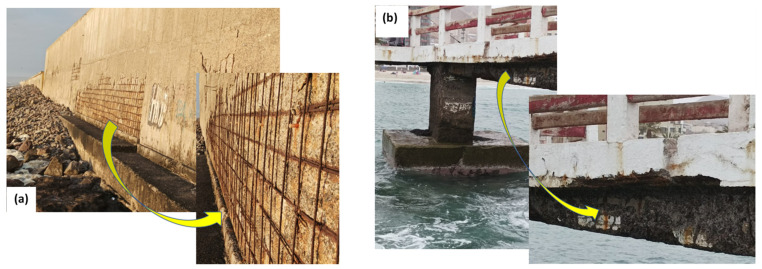
Damaged buildings after 10 years in contact with the marine environment, Iquique, Chile. Corrosion damages on (**a**) retaining wall structures and (**b**) bottom of the bridge.

The installation of seawater reverse osmosis (SWRO) plants is increasing to supply fresh water used by communities and for mineral processing, chemical industries, and, recently, green hydrogen production [1]. The corrosion of reinforcing steel exposed to seawater has received significant attention due to its widespread use in industrial and social infrastructures [2,3,4,5]. Multiple studies have demonstrated that corrosion initiation is a stochastic process that starts at random sites on steel surfaces and is highly susceptible to the concentration of chloride ions present in the solution [6,7,8,9]. Additionally, the service life of reinforced steels exposed to marine environments is affected by factors such as the infiltration of chloride and sulfate ions via diffusion or other mechanisms into the carbon steels [10,11]; the reaction between carbon dioxide and calcium hydroxide products of cement hydration, which generates calcium carbonate [9,10,11,12]; and unsatisfactory depth cover or excessive porosity in the concrete, which increases the risk of the penetration of gases and aggressive ions into the concrete [13,14,15,16,17]. Furthermore, other factors that affect the corrosion rate include the heterogeneity of the concrete and the type of carbon steel [18], the pH of the concrete pore water, the carbonation of the cement paste, the presence of cracks in the concrete, and galvanic effects due to contact between dissimilar metals [19,20,21]. These factors promote the corrosion of the steel–concrete interface, reducing the service life of the structure [6,22,23,24,25]. Figure 1 shows examples of buildings on the coast of Iquique, Chile, that have not been in operation for more than 10 years and have visual corrosion damage on the walls, slab foundation (Figure 1a), and at the bottom of the bridge (Figure 1b). These damages are associated with failures in the concrete, where cracks and detachments lead to the reinforced steel being directly exposed to the seawater, which is later corroded and accelerated by the high chloride ion content in the seawater.

The corrosion of reinforced steel caused by the penetration and accumulation of chloride ions into the reinforced concrete is called chloride-induced corrosion [6,9,13,26,27]. In this corrosion mechanism, the chloride ions ingress into the reinforced concrete structures through random parameters related to mass transport and the chemical properties of the material, generating unpredictable consequences on the surface of the steel [8]. The critical chloride concentration in concrete is also related to the oxygen content, which plays an important role in changes in the electrochemical potential [21,28]. Reinforced steel is vulnerable to generating corrosion microcells, especially when exposed to cycles of wetting and drying processes due to changes in the sea level during the day [15,26,29]. All these changes in reinforced concrete structures, which are linked to an alkaline environment, promote passive conditions (pH 12.5–13.8), creating a strong rust film that limits corrosion [30]. However, a high chloride concentration in the concrete structure promotes major internal corrosion sites, where the metallic deterioration is enhanced as a result of the internal forces generated by the brittle fracture of solid corrosion products that occupy a larger volume than the original volume of steel, generating cracks and significant seawater penetration [16]. Seawater penetration accelerates the corrosion rate by two orders of magnitude when the crack length exceeds 0.20 mm [16], decreasing the durability and resistance of the marine structure [6].

This research presents relevant information related to the corrosion behavior of A630-420H steel, which complements the published information on reinforced carbon steel in contact with saline media. The experimental potentiodynamic measurements were fitted to mathematical equations to determine the corrosion rates and kinetic parameters using a non-linear model based on mixed potential theory. These results were also compared with those obtained for other inexpensive carbon steels. The electrochemical findings were then correlated with the morphological and microstructural changes in the A630-420H steel. Notably, no corrosion studies have been conducted for A630-420H steel in saline solutions. Thus, the novelty of the present work lies in understanding the corrosion behavior of A630-420H steel when directly exposed to saline media. This study may provide valuable guidance for future corrosion studies on reinforced concrete structures.

## 2. Experimental Section

### 2.1. Electrochemical Measurements

The experimental procedure was designed to examine the kinetics of the partial electrochemical reactions in A630-420H steel immersed in 0.5 M NaCl solution, with a focus on the hydrogen evolution reaction (HER), oxygen reduction reaction (ORR), and iron oxidation reaction (IOR). For this purpose, A630-420H steel (CAP Acero, Compañia Siderúrgica Huachipato S.A., Talcahuano, Chile) with a nominal composition of (wt.%) 0.18–0.44 C, 0.6–1.1 Mn, ≤0.012 P, ≤0.04 S, ≤0.15 Si, and Fe as the balance was used. This steel has a maximum tensile strength and minimum yield strength of 630 and 420 MPa, respectively. To prepare the test solution, artificial seawater consisting of 0.5 M NaCl was made using analytical-grade NaCl salt (99.9% pure Sigma-Aldrich, Santiago, Chile) dissolved in deionized water.

**Figure 2:**
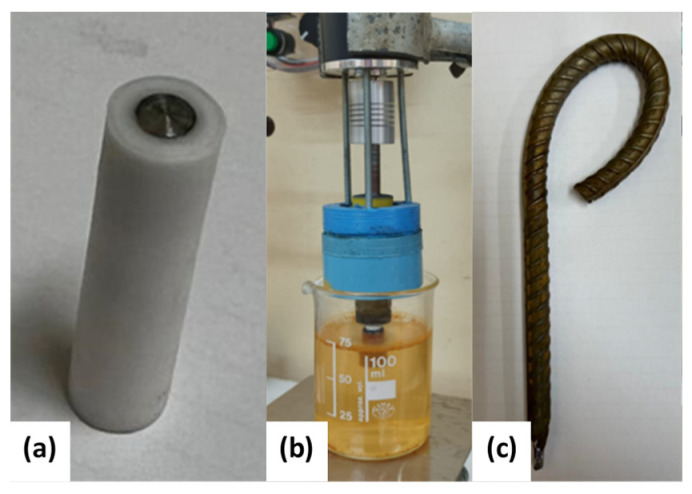
Electrode geometries for corrosion analysis made using 630A-420H steel: (**a**) rotating disc electrode for LSV measurements, (**b**) cylindrical electrode for WL measurements, and (**c**) bent electrode for LVS measurements under mechanical stress conditions.

Three different electrodes were prepared for this study, as shown in Figure 2, and are described as follows:

(i) Electrodes for linear sweep voltammetry (LSV) measurements: The electrodes were fabricated from a cylindrical rod of the specimen with a diameter of 4 mm and a length of 10 mm. The electrodes were concentrically inserted into a PTFE tube with a diameter of 8 mm and sealed with epoxy resin (Figure 2a). The electrochemical measurements were performed using a rotating disc electrode (RDE) interface at a rotation rate of 1200 rpm.
(ii) Electrodes for weight-loss (WL) measurements: The electrodes were cylindrical bars measuring 10 cm in length and 10 mm in diameter. The electrodes were immersed in the test solution under open circuit potential (OCP) conditions for 48 h while rotating at 1200 rpm (Figure 2b). The electrodes were designed with a threaded extension to be coupled to a motor shaft adaptor, and an O-ring seal was used to keep the threaded section dry while the steel specimen was immersed in the electrolyte. This electrode design prevents the occurrence of crevice corrosion and ensures that reliable weight-loss measurements can be obtained.
(iii) Electrodes to simulate mechanical stress conditions: The electrodes were fabricated using a 160 mm long and 6 mm diameter bar. The upper parts of these electrodes were bent to simulate the mechanical stress conditions experienced by construction bars (Figure 2c). The curved electrodes were exposed to the test solution for 288 h under OCP and stationary hydrodynamic conditions. Several LSV measurements were conducted during this period.

Before each experiment, the electrodes were sequentially polished using SiC paper of up to 1200 grit, washed with isopropanol, and rinsed with distilled water, except for the bent electrodes, which were studied as received. The temperature for all experiments was maintained at 20 ± 0.5 °C using a water-jacketed cell with circulating water through a thermoelectric temperature control device. All experiments were repeated in triplicate to verify their reproducibility. All potentials reported refer to a standard hydrogen electrode (SHE). The electrochemical analysis was performed using a rotating disc electrode interphase (RDE-2, BASi, West Lafayette, IN, USA) connected to a potentiostat/galvanostat (EPSILON, BASi, West Lafayette, IN, USA). LSV measurements were conducted at a scan rate of 2 mV/s in a conventional 3-electrode cell system using A630-420H steel as the working electrode (WE), Pt wire as the counter electrode (CE), and Ag/AgCl (4 M KCl sat.) as a reference electrode (RE). The experimental protocol for the polarization data was according to previous work [31] in a potential range between −1000 and 0 mV/SHE. Before the voltammetry measurements, the working electrode was maintained at −1000 mV/SHE for 30 s. Furthermore, the commercial carbon steels AISI 1020—with a nominal composition of (wt.%) 0.18–0.23 C, 0.3–0.6 Mn, 0.1–0.35 Si, ≤0.04 P, ≤0.05 S, and Fe as the balance—and A36—with a nominal composition of (wt.%) ≤0.29 C, 0.8–1.2 Mn, ≤0.4 Si, ≤0.04 P, ≤0.05 S, and Fe as the balance—were used to compare their corrosion behaviors with that of A630-420H steel. To determine the corrosion rate from the WL measurements, the electrodes were weighed before and after corrosion. After corrosion, the samples were washed in a 2% citric acid solution in an ultrasonic bath to remove the residual corrosion products, cleaned with deionized water and isopropyl alcohol, and finally dried with hot airflow.

### 2.2. Kinetic Analysis

The kinetic analysis was studied by applying non-linear fitting to the experimental polarization curves obtained from the LVS measurements. The superposition model, based on mixed potential theory, was utilized following the methodology described in our previous work concerning controls related to mass diffusion, charge transfer, and passivation mechanisms [31,32,33,34]. The following set of kinetic expressions (Equations (1)–(4)) is used as part of the non-linear fitting methodology, which considers the total current density (i) as a function of the partial current densities for the ORR ($i_{O_{2}}$), IOR ($i_{Fe}$), and HER ($i_{H_{2}}$):

(1) $$i=i_{O_{2}}+i_{H_{2}}+i_{Fe}\;$$

(2) $$i_{O_{2}}=i_{0,O_{2}}{\left(1-\frac{i_{O_{2}}}{i_{l,O_{2}}}\right)}^{m}e^{\left(\frac{-2.303\eta_{O_{2}}}{t_{O_{2}}}\right)}$$

(3) $$i_{H_{2}}=i_{0,H_{2}}e^{\left(\frac{-2.303\eta_{H_{2}}}{t_{H_{2}}}\right)}\;$$

(4) $$i_{Fe}=i_{0,Fe}e^{\left(\frac{2.303\eta_{Fe}}{t_{Fe}}\right)}$$

where $i_{0,O_{2}}$, $i_{0,H_{2}}$, and $i_{0,Fe}$ are the exchange current densities for the ORR, HER, and IOR, respectively; $i_{l,O_{2}}$ is the limiting current density for the ORR; $\eta_{O_{2}}=E-E_{eqO_{2}}$, $\eta_{H_{2}}=E-E_{eqH_{2}}$, and $\eta_{Fe}=E-E_{eqFe}$ are the overpotentials for the ORR, HER, and IOR, respectively; E is the electrochemical potential; and $E_{eqO_{2}}$, $E_{eqH_{2}}$, and $E_{eqFe}$ are the equilibrium potentials for the ORR, HER, and IOR, respectively. Finally, $t_{O_{2}}$, $t_{H_{2}}$, and $t_{Fe}$ are Tafel slopes for the ORR, HER, and IOR, respectively, and m is the kinetic order for the ORR and is equal to 0.5. The kinetic parameters were calculated from the fitting of Equations (1)–(4) to the experimental data.

### 2.3. Surface Analysis

The morphological characterization of the samples was performed using scanning electron microscopy (SEM) and energy-dispersive X-ray spectroscopy (EDS) using the following instruments: a Zeiss EVO MA 10 (Zeiss, Oberkochen, Germany), a Leo 1420p (LEO Electron Microscopy Ltd., New York, NY, USA), and an FE-SEM Hitachi model SU-5000 (Tokyo, Japan). The corrosion products layer was studied using X-ray diffraction (XRD) with a Shimadzu XRD-600 diffractometer (Shimadzu Corp., Kyoto, Japan) using Cu Kα radiation at an angular step of 0.02° (2θ) and counting time per step of 4 s.

## 3. Result and Discussion

### 3.1. Corrosion Analysis via Electrochemical Polarization

**Figure 3:**
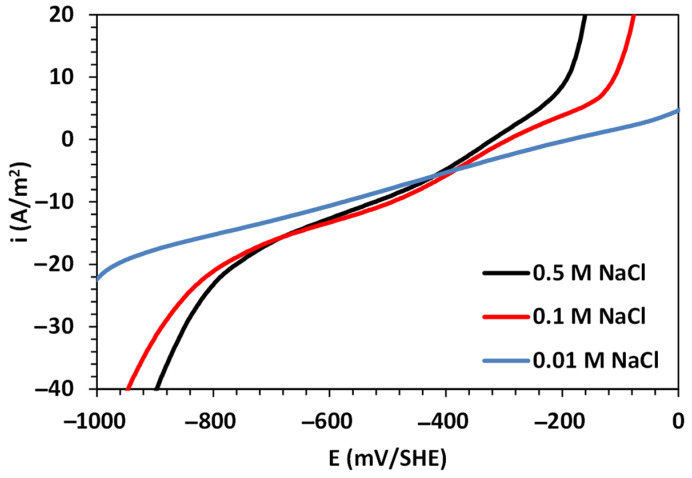
Linear polarization curves for A630-420H steel in aerated sodium chloride solutions.

**Figure 4:**
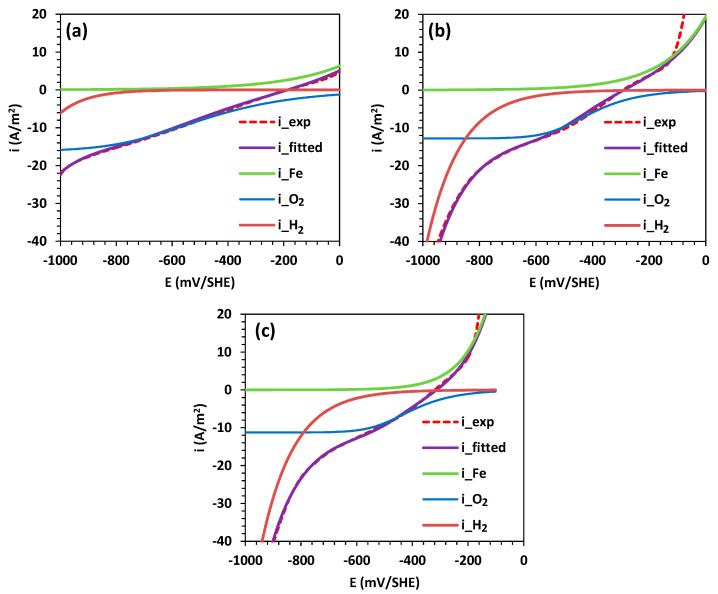
Deconstruction of polarization curves by applying mixed potential theory for A630-420H steel in contact with saline solutions: (**a**) 0.01 M NaCl, (**b**) 0.1 M NaCl, and (**c**) 0.5 M NaCl.

Figure 3 shows the electrochemical polarization curves for an A630-420H steel electrode immersed in a 0.5 M NaCl solution. The figure also includes the polarization curves obtained in 0.1 and 0.01 M NaCl solutions to compare the influence of chloride ions on the corrosion rate. Furthermore, Figure 4 shows the polarization curves for the partial HER, ORR, and IOR, which were formulated using Equation (1) through non-linear data fitting.

From the results shown in Figure 3, it is evident that the concentration of sodium chloride has a strong influence on the electrochemical response. The dissimilarity of the results is mainly reflected in the increased Tafel slope for both the cathodic and anodic branches, as well as the shifting of the corrosion potential ($E_{corr}$) in the cathodic direction. On the one hand, these shifts in $E_{corr}$ values are related to the variations in the physical properties of the test solution, which promote lower oxygen solubility with an increase in solution salinity. On the other hand, the increases in the anodic and cathodic branches with the sodium chloride concentration are related to the high activity of chloride ions and their diffusion on the metallic surface. This leads to an increase in the kinetics of the IOR and ORR, ultimately promoting high corrosion rates.

The analysis of the polarization data for A630-420H steel in contact with NaCl solutions has demonstrated that mixed potential theory can be successfully applied to decompose the total current into a linear contribution of the partial electrochemical HER, ORR, and IOR, as shown in Figure 4 [31,33,34,35]. While the partial HER and IOR follow a charge transfer kinetic mechanism, the ORR follows a kinetic expression under a mixed mechanism of charge transfer and diffusion control. The obtained kinetic and corrosion parameters are tabulated in Table 1. As mentioned in the previous section, the anodic branch increased with the NaCl concentration, which is consistent with the calculated anodic Tafel slope values and the trends observed in the ORR parameters, where reductions in the exchange current density and limiting current density were observed. These variations resulted in high corrosion rates, reaching a value of 3.02 A/m^2^ for 0.5 M NaCl, indicating a high susceptibility to pitting corrosion. This is also evident in the abrupt increase in the current density at potentials close to −100 mV/SHE. Furthermore, the results reveal higher activity for the HER with an increase in the NaCl concentration, as indicated by the increase in the exchange current density values.

**Figure 5:**
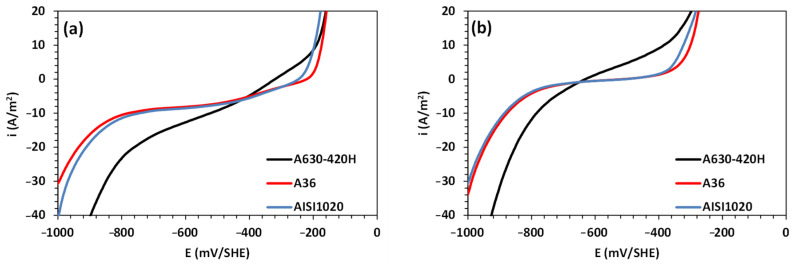
Linear polarization curves for A630-420H, A36, and AISI 1020 sheets of steel in 0.5 M NaCl solution under (**a**) aerated and (**b**) de-aerated conditions.

In the context of the corrosion behavior of steel, a comparative analysis of the corrosion behavior was performed for steel samples of types A36, AISI 1020, and A630-420H in an aerated and de-aerated 0.5 M NaCl solution, as shown in Figure 5. The oxygen content in the solution was purged with nitrogen for the measurements in the de-aerated solution. The results indicate similar electrochemical responses for AISI 1020 and A36 steels, contrasting with A630-420H, which shows significant differences in the cathodic branch and the region close to the inversion potential. Additionally, in both aerated and de-aerated solutions, the polarization curves reveal higher anodic and cathodic activities for the reinforced A630-420H steel, indicating a higher corrosion rate. This difference in corrosion rates can be attributed to the chemical composition of A630-420H steel, particularly its carbon and silicon content. The high carbon content promotes the presence of the perlite phase, leading to the formation of galvanic cells between the ferrite and cementite phases, thus increasing the corrosion rate. Furthermore, the addition of silicon promotes the formation of a denser and more compact corrosion products layer, which in turn limits the diffusion of aggressive ions on the metallic surface [36,37].

Table 2 presents the kinetic and corrosion parameters obtained from the experimental polarization data fitted with the mixed potential model in Equation (1). In Table 2, it can be observed that the corrosion potential for A630-420H steel is significantly shifted toward negative values compared with other common carbon steels, both in aerated and de-aerated conditions. Additionally, A630-420H steel exhibits higher exchange current densities for the corrosion ORR, HER, and IOR compared with A36 and AISI 1020 carbon steels. These variations and trends are also observed in both aerated and de-aerated solutions. Notably, the HER plays a significant role in the corrosion of A360-420H steel in near-neutral solutions, wherein the material is at a high risk of hydrogen introduction and diffusion into its matrix near the corrosion potential. This process can lead to embrittlement, which is characterized by severe internal damage in the form of blisters and hydrogen-induced crack formation [38].

### 3.2. Corrosion Analysis via Weight-Loss Measurements

The weight-loss measurements for A630-420H steel involved immersing it in a 0.5 M NaCl solution at a rotation rate of 1200 rpm for 48 h. The results show a weight loss of 0.35%, which corresponds to 56.5 g/m^2^. Calculating the corrosion current density equivalence using Faraday’s law, the weight loss magnitude was determined to be 1.16 A/m^2^. This value is lower than that obtained from linear voltammetry but higher than what was observed for AISI 1020 and A36 carbon steels. These variations in corrosion behavior can be attributed to the formation of an oxide film during the corrosion process, which partially restricts the diffusion of oxygen and chloride species on the metal surface.

**Figure 6:**
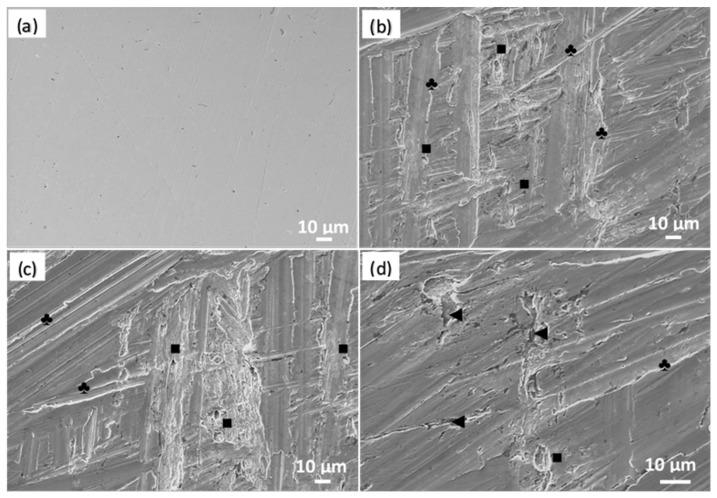
SEM images of A630-420H steel (**a**) before and (**b**–**d**) after corrosion process in aerated 0.5 M NaCl solution.

Figure 6 depicts the SEM images of the A630-420H steel electrode before and after immersion in the 0.5 M NaCl test solution. As a reference, the polished sample (Figure 6a) displays small scratches resulting from the manual polishing process and minor imperfections that do not correspond to pitting sites. Examining the SEM images of the samples after the corrosion process (Figure 6b–d), it is evident that the electrodes were entirely coated with an oxide film exhibiting various morphological patterns. These patterns also vary depending on the hydrodynamic conditions and surface treatment. The oxide film exhibits the formation of random lines (marked with the symbol ♣ in the figure) along with small pores (symbol ■ in the figure) and cracks (symbol ◄ in the figure). These imperfections (pores) and damages (cracks) are commonly observed in solutions containing halide ions such as chloride, which play a role in the anodic sub-process and promote pitting corrosion.

### 3.3. Corrosion Analysis under Mechanical Stress Conditions of the Electrode

**Figure 7:**
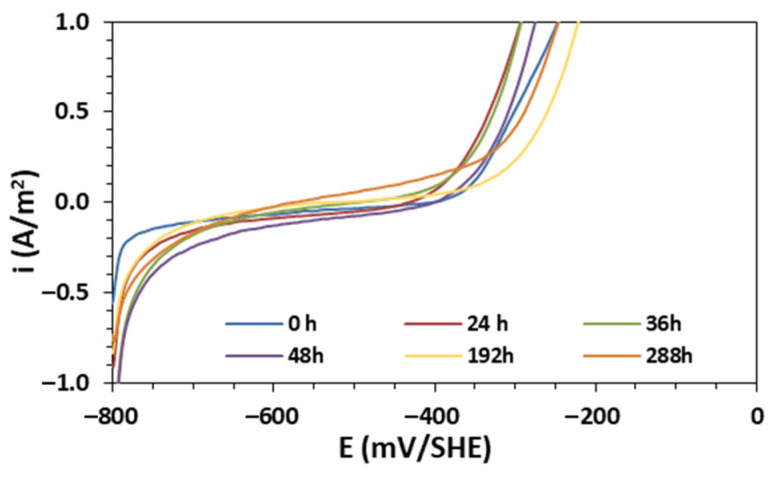
Linear polarization curves for A630-420H in contact with 0.5 M NaCl at OCP measured at different immersion times up to 288 h.

In these experiments, the corrosion of a bent A630-420H electrode immersed in an aerated 0.5 M NaCl solution for 288 h under OCP and stagnant hydrodynamic conditions was investigated. Figure 7 illustrates the electrochemical measurements of the electrode over time, which were recorded using linear sweep voltammetry to gain a better understanding of the action of the corrosion products formed on the metallic surface.

The results show that as time progresses, the polarization curve shifts towards cathodic potentials in a potential range between −400 and −600 mV/SHE. As expected, these findings are associated with the formation of a corrosion products layer on the metallic surface as the corrosion process advances. This corrosion layer restricts the mass transfer of dissolved oxygen from the bulk to the metallic surface, resulting in competition between the metal dissolution reactions, iron oxide transformation, and further precipitation [32]. Consequently, the potentiodynamic curves exhibit a decrease in the planar plateau at intermediate cathodic potentials (between −650 and −450 mV/SHE) after 36 h of immersion, indicating a progressive increase in the cathodic exponential branch attributed to the HER. Subsequently, for immersion times of longer than 36 h, the HER contributes more significantly to the corrosion rate compared with the ORR.

**Figure 8:**
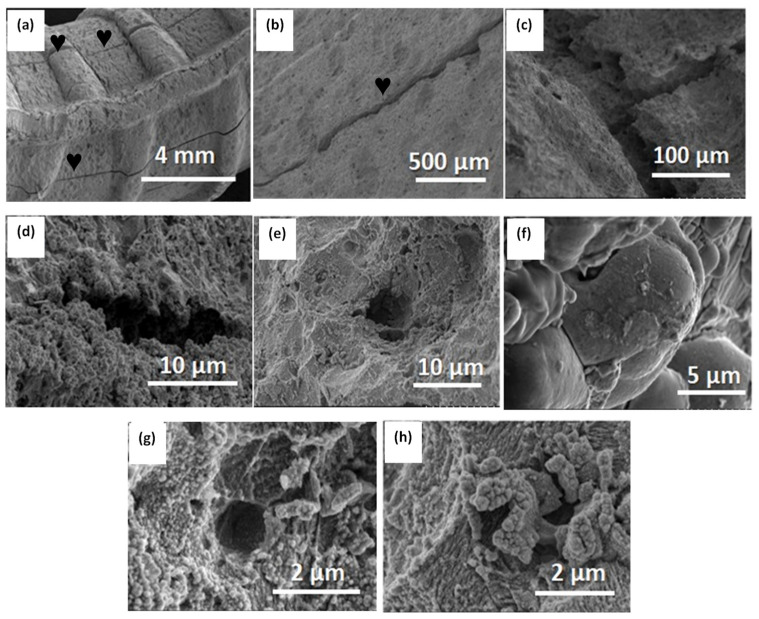
SEM images of corroded A630-420H steel after 288 h of immersion in 0.5 M NaCl solution with formation of (**a**–**d**) cracks and (**e**–**h**) pore sites.

Figure 8 depicts SEM images of the corroded steel sample, providing significant information about the electrochemical attack and evident deterioration on the metallic surface. Upon general inspection of the images shown in Figure 8, noticeable deterioration of the steel sample and the formation of a robust corrosion products film on the surface can be observed. Additionally, Figure 8a shows a localized brittle fracture of the corrosion products layer oriented longitudinally in the direction of the bent section of the steel sample, resulting in the formation of cracks (symbol ♥ in the figure). Figure 8b–d provide a more detailed examination of the crack formed on the corrosion products layer, where mechanical, chemical, and electrochemical effects are involved, including mechanical stress, the expansion of the corrosion products layer due to the transformation of iron oxides, and the evolution of molecular hydrogen during the cathodic subprocess. The morphology of the crack reveals dimensions of 16.6 μm in length and 3.66–3.99 μm in width, with a separation of 23.2 to 31.5 μm. Additionally, this steel exhibits pore sites with cylindrical aspects that are prone to retaining the aggressive solution, thereby accelerating iron dissolution during the anodic subprocess, which is further enhanced by the presence of chloride. In general, the pores’ geometry exhibits well-delineated contours with sizes close to 1.10 μm, surrounded by corrosion products, as shown in Figure 8e–h.

**Figure 9:**
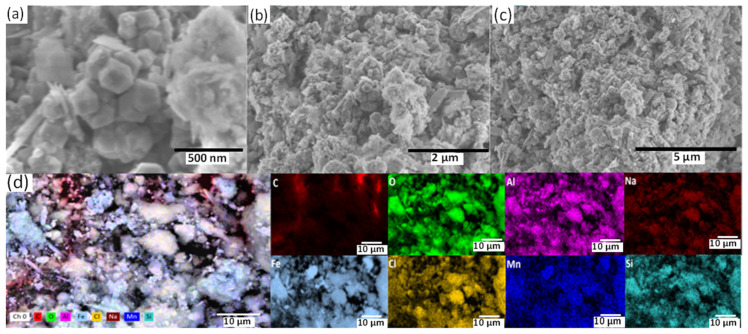
SEM images of the corrosion products formed on the A630-420H steel surface after 288 h of immersion in 0.5 M NaCl solution, (**a**) magnification of 70,000×, (**b**) magnification of 20,000×, (**c**) magnification of 10,000×, and (**d**) elemental mapping of the corrosion products at magnification of 1999×.

Furthermore, to gain a comprehensive understanding of the corrosion of A630-420H steel, elemental mapping of the corrosion products is presented in Figure 9. Based on the results, it can be deduced that the corrosion products are composed of iron oxides. However, the presence of chlorine can be attributed to the formation of NaCl during the drying process of the sample. Additionally, the presence of aluminum and silicon may be associated with their respective oxides, which are impurities that were present in the electrode since it was used as received.

**Figure 10:**
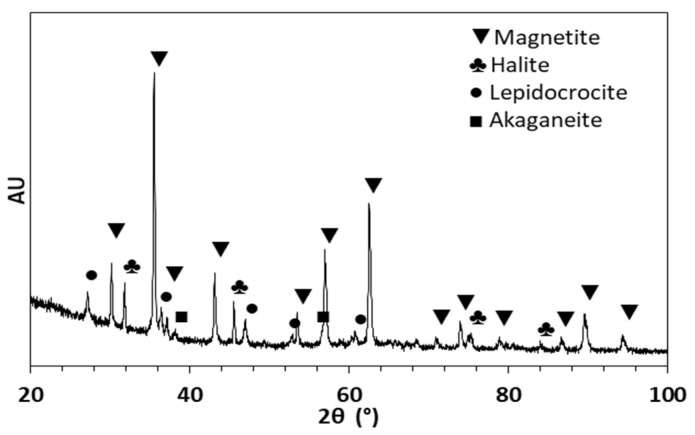
XRD patterns for corrosion products after corrosion process of A630-420H samples immersed in 0.5 M NaCl solution during 288 h.

Figure 10 shows an XRD analysis of the corrosion products produced by the corrosion of A630-420H steel after 288 h of exposure.

The XRD results provide evidence of well-known patterns attributed to oxides such as Fe_3_O_4_ (magnetite) and oxyhydroxides such as γ-FeOOH (lepidocrocite) and β-FeOOH (akaganeite). The presence of the akaganeite phase indicates the transformation of a previous green rust phase [Fe_3_^2+^Fe^3+^(OH)_8_]^+^[Cl·nH_2_O]^−^ (hydroxychloride) into akaganeite due to the prolonged exposure times of the sample in a high-chloride-concentration solution [39]. Additionally, the presence of the magnetite phase is consistent with the polarization curves, such that a decrease in the dissolved oxygen near the metallic surface leads to a low-oxygen-concentration environment in which magnetite remains stable [32]. Lastly, the halite (NaCl) phase is attributed to the drying process of the sample and the crystallization of the aqueous NaCl present in the test solution.

## 4. Conclusions

This study presented the corrosion behavior of A630-420H carbon steel in NaCl solution. The electrochemical measurements revealed significantly high corrosion rates in chloride solutions, reaching up to 3.02 A/m^2^ for the 0.5 M NaCl solution. These corrosion rates were higher than those obtained for the AISI 1020 and A36 carbon steel samples in both aerated and de-aerated solutions. The corrosion mechanism is strongly associated with changes in the cathodic reaction, where the hydrogen evolution reaction plays a significant role at high chloride ion concentrations in the solution. The influence of chloride ions on the corrosion of A630-420H steel was indicated in the sudden increase in the current density of the anodic branch, demonstrating the low resistance of this steel to chloride ion attack. The weight-loss measurements revealed a lower corrosion rate compared with the electrochemical measurements, equivalent to 1.16 A/m^2^. This fact is associated with the formation of an oxide film on the surface as a corrosion product, which limits the diffusion of oxygen and chlorides on the metallic surface. This condition was verified via SEM analysis, which determined that an oxide film was deposited on the corroded metallic surface, showing the presence of pores and cracks. Furthermore, for A630-420H steel under mechanical stress conditions, the data demonstrated that the hydrogen evolution reaction became more relevant in the corrosion behavior for immersion times longer than 36 h. Under these conditions, the steel exhibited significant morphological damages, including a localized brittle fracture of the corrosion products layer oriented longitudinally in the direction of the bend section. These damages were further enhanced by the presence of pores and cracks. The XRD analysis indicated that the oxide film was mainly composed of magnetite and lepidocrocite. The high sensitivity of A630-420H carbon steel to corrosion in saline environments defines the novelty of this contribution, which may provide valuable guidance for future corrosion studies.

## Figures and Tables

**Table 1:** Kinetic and corrosion parameters calculated from experimental polarization curves for A630-420 H steel immersed in sodium chloride solutions.

Parameter	NaCl, M		
	0.01	0.1	0.5
$i_{0,Fe}$, A/m^2^	0.34	0.32	0.10
$t_{Fe}$, mV/SHE	490	349	212
$i_{0,O_{2}}$, A/m^2^	−3.27 × 10^−2^	−1.62 × 10^−4^	−7.35 × 10^−5^
$t_{O_{2}}$, mV/SHE	−525	−263	−247
$i_{l,O_{2}}$, A/m^2^	−16.2	−12.8	−11.3
$i_{0,H_{2}}$, A/m^2^	−0.01	−0.29	−0.39
$t_{H_{2}}$, mV/SHE	−228	−276	−270
$E_{corr}$, mV/SHE	−186	−293	−320
$i_{corr}$, A/m^2^	2.63	2.80	3.02

**Table 2:** Kinetic and corrosion parameters were calculated from experimental polarization curves for A630-420H, A36, and AISI 1020 steels in 0.5 M NaCl solution under aerated and de-aerated conditions.

Parameter	Steel Type in Aerated 0.5 M NaCl			Steel Type in De-Aerated 0.5 M NaCl		
	A36	AISI 1020	A360-420H	A36	AISI 1020	A360-420H
$i_{0,Fe}$, A/m^2^	1.46 × 10^−7^	2.96 × 10^−6^	0.10	9.41 × 10^−3^	0.01	2.33
$t_{Fe}$, mV/SHE	57	65	212	107	104	358
$i_{0,O_{2}}$, A/m^2^	−5.69 × 10^−5^	−3.35 × 10^−5^	−7.35 × 10^−5^	-	-	-
$t_{O_{2}}$, mV/SHE	−240	−227	−247	-	-	-
$i_{l,O_{2}}$, A/m^2^	−8.1	−8.3	−11.3	-	-	-
$i_{0,H_{2}}$, A/m^2^	−0.02	−0.03	−0.39	−0.06	−0.05	−0.25
$t_{H_{2}}$, mV/SHE	−196	−198	−270	−215	−211	−238
$E_{corr}$, mV/SHE	−221	−247	−320	−492	−507	−625
$i_{corr}$, A/m^2^	1.36	1.74	3.02	0.15	0.16	2.29

## Data Availability

The supporting data are available from the corresponding authors.
